# P-2220. Real-World Utilization of a Multiplex Pneumonia PCR Panel on Antimicrobial Stewardship and Antibiotic Use

**DOI:** 10.1093/ofid/ofae631.2374

**Published:** 2025-01-29

**Authors:** Elizabeth Keil, Alyssa Chionglo, Javeed Siddiqui

**Affiliations:** Providence Queen of the Valley Medical Center, Napa, California; Providence Queen of the Valley Medical Center, Napa, California; TeleMed2U, Carlsbad , CA

## Abstract

**Background:**

In October 2022 the Queen of the Valley Medical Center (QVMC) microbiology department implemented the Biofire® Pneumonia Pathogen Panel polymerase chain reaction (PCR) for identification of viral and bacterial pathogens along with resistance genes. This may aid in improved identification of pneumonia pathogens with a quicker turnaround time. In contrast, respiratory cultures take multiple days to return with both the organism and antibiotic susceptibility, which delays the time to achieving targeted therapy.

**Methods:**

This was a retrospective cohort study from October 2022 to April 2023. Patients were included if they were admitted to QVMC and had a positive pneumonia PCR. Patients were excluded if they were receiving antibiotics for an indication other than pneumonia, if they did not receive antibiotics, or if they were discharged, transferred or died prior to the PCR result. The primary outcome was if the patient achieved targeted therapy. Secondary outcomes included time to targeted therapy, actions performed based on the results, and correlation between respiratory culture results and PCR.

**Results:**

Fifty-nine patients were included for analysis. Most patients had a diagnosis of CAP (n = 43, 72%), vs. HAP (n = 8, 14%), and VAP (n = 8, 14%). Targeted therapy was achieved in 41 patients (70%). Seven (12%) of these patients had empiric therapy which was also targeted therapy. The mean time from PCR collection to the start of targeted therapy was 23.11 hours. For patients who had respiratory cultures collected, the mean time to final identification with susceptibilities was 40.6 hours. Therefore, targeted therapy was achieved at least 17 hours sooner than if de-escalation or escalation was solely dependent upon respiratory culture results. There were 47 antimicrobial stewardship recommendations documented, and 31 (66%) were accepted by providers. Forty-four patients (75%) also had respiratory cultures collected, and only 19 (43%) had organism growth.

**Conclusion:**

Utilizing multiplex PCR technology for patients with pneumonia can be useful for antimicrobial stewardship programs by providing higher rates of pathogen identification and resistance data compared to respiratory culture alone. This allows for improved time to targeted therapy.

**Disclosures:**

All Authors: No reported disclosures

